# Effective dispersal of fern spore and the ecological relevance of zoochory

**DOI:** 10.1111/brv.70038

**Published:** 2025-05-29

**Authors:** James M. R. Brock

**Affiliations:** ^1^ University of Auckland Private Bag 92019 Auckland 1142 New Zealand

**Keywords:** wind, animal, bird, pteridophyte, biogeography, speciation, island, metapopulation, isolation, landscape ecology

## Abstract

The mechanisms of fern dispersal are under‐studied and there are few data to support the vectors assumed to drive patterns of sporophyte occurrence and speciation. Although wind is generally the fern spore dispersal vector described in the literature, there has always been competing evidence supporting alternate vectors. Despite this, established patterns of dispersal are generally discussed in the context of wind. The assumptions around wind as a dispersal vector and the possibilities of single‐spore establishment have confounded discussions on effective dispersal of fern spore, fern meta‐population dynamics, and fern speciation. In this study, I review (*i*) the importance of spore load across taxa, (*ii*) evidence for vectors of fern spore, (*iii*) the environmental tolerances of fern life stages, and (*iv*) the relevance of, and constraints on, different dispersal vectors in the context of increasingly hazardous landscape matrices. I conclude that whilst wind is an important dispersal vector in non‐hazardous landscapes, directed dispersal by an animal vector to isolated safe sites in a hazardous landscape matrix may be key for fern metapopulations and communities.

## I. INTRODUCTION

Dispersal determines connections across and within plant populations, speciation events, and community dynamics at local and landscape scales (Claramunt *et al*., 2011; Török *et al*., 2020; Denk & Hallatschek, 2022). Dispersal has been considered extensively in the scientific literature for embryophytes (Eriksson & Jakobsson, 1999; Thomson *et al*., 2011); however, little research has been published on pteridophyte dispersal (Rose & Dassler, 2017), and the small size of fern spore has driven assumptions that wind is the dominant or sole effective disperser of ferns (Ridley, 1930; Polunin, 1960; Tryon, 1970; Sessa *et al*., 2015; Green, Baltzinger & Lovas‐Kiss, 2022). While anemochory is considered the vector of fern spore (Tryon, 1986; Page, 2002; Geiger *et al*., 2007), surprisingly little research has examined this hypothesis (Page, 2002). Furthermore, there is increasing evidence that zoochory also contributes to fern spore movements (Boch *et al*., 2016; Lovas‐Kiss *et al*., 2018); however, the extent to which zoochory is important as a vector remains unqualified and unquantified.

Wind was suggested as a key vector of fern spore as early as 1880 (Wallace, 1880), but the first considerations around fern biogeography were published by Tryon (1966, 1970, 1986). Tryon (1970) commented on the strong similarity of the fern flora of Madeira and the Azores Islands, over 800 km apart, and described insular fern flora as a subset of the adjacent continental landmass, with occasional “waif species” from more distant sites. Although alternative hypotheses suggest that environmental filters affecting the development of any deposited spore are more significant than dispersal potential (Christ, 1910; Pettersson, 1940), and that trade winds oppose the necessary direction of travel to explain the relationships between continental and insular fern flora such as on Fiji and Saint Helena (Copeland, 1929; Muir & Baker, 1968), wind remains the vector most commonly associated with fern dispersal (Page, 2002). Yet the patterns of species distribution that drove the ideas around ready long‐distance wind dispersal of fern spore are changing. Acknowledging the limitations imposed by reticulate evolution (Sessa *et al*., 2015), recent phylogenetic research suggests that vicariance has played a significant role in the distribution of modern fern taxa, suggesting constraints on the ubiquitous and ready nature of fern spore dispersal (Der *et al*., 2009; Hennequin *et al*., 2010; Sessa, Zimmer & Givnish, 2012; Noben *et al*., 2017; Testo *et al*., 2022). For example, *Pyrrosia serpens* (G.Forst.) Ching, which was given as an example by Tryon (1970) as a broadly distributed species across Australia and New Zealand, has since been split into at least two separate species with much reduced ranges (Hovenkamp, 1986). Further, various authorities have suggested that species aggregates and taxa with large distributions require attention using modern molecular techniques to establish whether these taxa are instead a number of distinct species, for example *Ophioglossum coriaceum* A.Cunn. agg., and *Rumohra adiantiformis* (G.Forst.) Ching (Wardle *et al*., 2001; de Lange & Rolfe, 2010).

Beyond similarities across insular fern communities, a second pattern suggesting that some fern taxa can disperse readily is the over‐representation (disharmony, *sensu* Carlquist, 1966) of ferns in insular flora (Tryon, 1970, 1986; Kreft *et al*., 2010). By contrast, orchids, with similarly minute, wind‐dispersed propagules, are significantly under‐represented on islands, suggesting that distal factors such as abiotic filtering, facilitation (or a lack of mycorrhizae in this example), and competition may preclude the establishment of otherwise readily dispersed taxa in islands (Taylor *et al*., 2019). Therefore, for ferns to be over‐represented on islands, fern spore must be more successful in dispersing to islands in comparison to other taxa (in terms of abundance and frequency of arriving viable propagules), and also in establishing (depositing in suitable environmental conditions; escaping competition/predation; viable populations establishing; Hille Ris Lambers *et al*., 2012). A further complexity in fern disharmony is that the proportional representation of ferns with chlorophyllous spores (short‐lived spore with active chloroplasts recorded in 441 species, or 17% of fern species with known spore colour; Mellado‐Mansilla *et al*., 2021), is highest in island floras (D. Mellado‐Mansilla, P. Weigelt, M. Kessler, D. Craven, G. Zotz & H. Kreft, in preparation). The findings of Mellado‐Mansilla *et al*. (in preparation) raise questions around spore vectors, as chlorophyllous spores are understood to be limited by wind dispersal due to their lower desiccation tolerance (López‐Pozo *et al*., 2019).

The ability of ferns to establish at a new site from a single spore is significant in the wind dispersal literature (Tryon, 1970; Geiger *et al*., 2007). Yet, although single‐spore colonisation may occur through gametophytic selfing or through apomixis (de Groot *et al*., 2012; Liu *et al*., 2012; Grusz, 2016; Sessa, Testo & Watkins, 2016), the likelihood of single‐spore colonisation has been questioned (Soltis & Soltis, 1992; Ranker & Haufler, 2008; Haufler *et al*., 2016). Gametophytic selfing is possible for many fern species under experimental conditions (Sessa *et al*., 2016), but ferns generally promote out‐crossing and naturally occurring homosporous fern populations have been shown to be the product of out‐crossing (Haufler & Gastony, 1978; Ranker & Geiger,^2008^). Studies that have followed sporophytes produced by gametophytic selfing over time have shown that, when compared to sporophytes produced by out‐crossing, selfed plants experienced significantly higher rates of gross developmental abnormalities, stunted growth, and mortality, so the potential for, and effectiveness of, single‐spore colonisation is uncertain (Peck, Peck & Farrar, 1990; Schneller & Holderegger, 1997). Further, the constraints on single‐spore colonisation events should be considered in the context of (*i*) spore viability in the atmosphere [an issue raised first by Wernich (1880) and investigated by van Overeem (1937) and Gradstein & van Zanten (1999)], (*ii*) gametophyte persistence (e.g. Farrar & Mickel, 1991; Testo & Watkins, 2011), and (*iii*) successful reproduction (Christ, 1910).

What of possible alternative vectors to wind? Fern zoochory has been identified across a range of animals including invertebrates, amphibians, and mammals (Oralls, Osborn & Tessier, 2016; Brock & Collier, 2020), and birds in particular have been highlighted as potential fern dispersers (Wallace, 1880; Boch *et al*., 2016; Abeyrama *et al*., 2021). Successful endozoochory has been shown for spore, sporangia, and sections of sporophyte, and potential evidence of epizoochory exists in the presence of spore material in feathers and fur (Wallace, 1880). Further, palaeoecologists have recorded fern spores and sporangia in coprolites and fossilised gut material of dicynodonts [235 million years ago (Mya); Fiorelli *et al*., 2013] and nodosaurs (145–100 Mya; Brown *et al*., 2020), showing that ferns, as embryophytes, may have evolved with animals as vectors. However, as with wind, the effectiveness of animals as a vector of fern spore has never been established and the context in which animal dispersal may be significant in supporting the distribution patterns observed globally are generally overshadowed by the assumed broad potential of anemochory (Pettersson, 1940; Boch *et al*., 2016).

An additional dispersal propagule for ferns is their gametophytic gemmae (Farrar, 1985; Parris, 2001). Gemmae are multi‐functional structures that potentially function as propagules and, once independent of the gametophyte, can develop antheridia and engage in sexual reproduction (Emigh & Farrar, 1977). Many fern species with chlorophyllous spore produce gemmae, perhaps providing an alternative propagule for these otherwise presumed dispersal‐limited species (Dassler & Farrar, 1997; Mellado‐Mansilla *et al*., in preparation). As propagules, gemmae potentially support both asexual reproduction and the ability of a fern to establish new gametophytes in a location other than where the parent spore germinated (Dassler & Farrar, 2001), and may also serve to support dispersal from gametophyte‐only populations (Ebihara *et al*., 2013). Again, little work has been undertaken to examine the effectiveness of gametophytic gemmae as a vector; the only published work on gemmae and dispersal is a genetic study of potential clones of the underground gametophyte of *Botrychium pumicola* Coville ex Underw., which suggests that subterranean gemmae do not contribute to fern dispersal (Camacho & Liston, 2001).

The aims of this review are to establish precisely what is known on fern spore dispersal, to encourage a discussion around spore dispersal in the context of landscape ecology, and to diversify the scientific narrative on fern dispersal. To achieve these aims, I review the following aspects of spore dispersal: (*i*) propagule pressure or spore load (*sensu* Rose & Dassler, 2017); (*ii*) evidence of vectored dispersal of fern spore; (*iii*) environmental tolerances of different fern life stages; and (*iv*) constraints of landscape heterogeneity on the effectiveness of spore vectors.

## II. PROPAGULE PRESSURE OR SPORE LOAD

The potential for taxa to disperse successfully increases with increasing propagule pressure (Heinrichs & Pauchard, 2015; Rose & Dassler, 2017). Inter‐specific spore production in ferns varies by orders of magnitude (Ridley, 1930; Peck *et al*., 1990), and spore production across individual plants is constrained to a single pulse of spores per frond per year (Rose & Dassler, 2017). Further, spore ornamentation, sporophyte population size, growth habit, and ecosystem associations all vary significantly across fern taxa. To compare propagule pressure (spore load *sensu* Rose & Dassler, 2017) as a driver of dispersal potential we must consider the following aspects: spore production, spore ejection, and fern habit.

### (1) Spore production

Estimates of annual spore load, based on the number of spores per frond, and fertile fronds per plant per annum, range between 54,000 spores per plant for *Cryptogramma stelleri* (Peck *et al*., 1990), and 18.9 billion for *Sphaeropteris cooperi* (Hook. ex F.Muell.) R.M.Tryon (Durand & Goldstein, 2001). Spore production per individual will be affected by population structure, driving variation in spore load across taxa; for example fern taxa that are diminutive (<50 cm), and occur at low densities (<50 individuals ha^−1^) such as *Ophioglossum* spp. produce spore loads that are orders of magnitude less than those of *Pteridium* species, which can establish as the dominant vegetation cover over several hectares (Wagner, Wagner & Beitel, 1985; McGlone, Wilmshurst & Leach, 2005). Further, seasonal variation in moisture availability and disturbance events, such as hurricane‐induced canopy damage, can influence investment and drive inter‐annual differences in fertile frond and spore production (Sharpe, 2022).

### (2) Spore ejection

Spore ejection from sporangial tissue and away from the parent sporophyte is achieved by a range of mechanisms from spore cavitation (Hovenkamp *et al*., 2009) and ejection of sporangia (Conant, 1978; Koller & Scheckler, 1986), to cavitating annuli that eject spores (Llorens *et al*., 2016). Spore release speeds range from 3 to 10 m s^−1^ (Poppinga *et al*., 2015; Llorens *et al*., 2016); in comparison, fungal spore ejection speeds range between 2 and 25 m s^−1^ (Yafetto *et al*., 2008). Studies from the USA, Spain, and the Azores show that release events appear to correlate with seasons of lower humidity and higher temperatures, and it is suggested that these conditions promote dispersal (Pettersson, 1940; Benedict, 1963; Arosa *et al*., 2009; Rodríguez De La Cruz, Sánchez Reyes & Sánchez Sánchez, 2009; Savage *et al*., 2012; Suissa, 2022). However, in regions with continuous water availability, for example in cloud forests, spore rain can continue year‐round suggesting that spore release processes vary with biome and autecology of different species and have phenological links to the gametophyte life stage (Gómez‐Noguez *et al*., 2017b; Quinlan *et al*., 2022).

The ballistics of microscopic particles describe how spore material decelerates rapidly after ejection (Pringle *et al*., 2017). The effects of air viscosity increase linearly with decreasing spore size, so that smaller spores will decelerate faster, although smaller spores have significantly less inertia which permits their persistence in the air column (Vogel, 2005; Fischer *et al*., 2010). Spore velocity in air columns is also affected by surface ornamentation such as ridges and grooves (Gómez‐Noguez *et al*., 2017a). Spore shape, mass, and ornamentation will therefore have a significant influence on the potential for spore material to escape the parent sporophyte (Pringle *et al*., 2017). Boundary layers – where moving air meets a structure, and friction drives a static layer of air over the surface of the structure (Pringle *et al*., 2017) – around the parent sporophyte will constrain the potential dispersal of spore and may result in the deposition of spore material adjacent to the parent sporophyte (Conant, 1978). However, the spore ejection process should drive an unknown proportion of the spore into air flows beyond the sporophyte.

### (3) Fern habit

Sporangia vary in their position on fronds across fern taxa and this will have varying effects on the trajectory of spores after release. The tree fern *Alsophila colensoi* Hook.f. has a prostrate trunk and the fronds are rarely 1 m above the forest floor; even though this taxon, as a tree fern, produces a large number of spores, most of this spore will likely be ejected onto the forest floor beneath the fern (Rose & Dassler, 2017; de Lange, 2021). As well as size, location, and relative position of the fertile structures of the sporophyte, the habit (terrestrial/epiphyte/aquatic) will also change the release conditions and effectiveness of dispersal. For example, the forest canopy functions as a filter, reducing the ability of understorey fern spore to escape into air currents and move away from the parent sporophyte (Gómez‐Noguez *et al*., 2017b). In comparison, small epiphytic ferns in the canopy of a forest, even though they may have a relatively small spore load, may have a significantly higher chance of distant spore dispersal than a tree fern in the forest understorey (Conant, 1978; Rose & Dassler, 2017).

Where plants are concealed by surrounding vegetation (i.e. where escaping the canopy is fundamental to dispersal; Nathan *et al*., 2002), such as some *Ophioglossum* spp., the spores are significantly less likely to escape the immediate deposition zone around the sporophyte than are those of, for example *Pteridium* species, which can grow to 6 m tall, are dominant in the landscape, or grow on the margins of woody vegetation and so are exposed to wind currents (Wagner *et al*., 1985; McGlone *et al*., 2005). Raynor, Ogden & Hayes (1976) tested lateral movement of fern spore in forests by experimentally releasing spore of *Dryopteris* (ovoidal, rough, 33–45 μm diameter) and *Osmunda* (spheroidal, smooth, 54 μm) from a height of 1.75 m and recorded spore no further than 40 m from the release point. Adaptation to the suppressive effect of the forest canopy on spore dispersal has been observed in *Onoclea sensibilis* L. which retains a fertile frond over winter and releases spore the following spring before canopy trees leaf in less humid conditions (Pettersson, 1940; Benedict, 1963; Suissa, 2022).

A key limitation on the escape of a spore from parent sporophyte adjacency is the presence of a boundary layer around the sporophyte, and over the adjacent vegetation. Although the constraints of boundary layers may result in the significant deposition of spore material around the parent sporophyte, the ejection process should drive a small proportion of the spore material into air flows beyond the sporophyte. Subsequent deposition of spore will be affected by boundary layers around the surrounding vegetation, spore size and ornamentation, and local air currents (Gómez‐Noguez *et al*., 2017a); recorded air‐speeds in the forest understorey are relatively low, e.g. 0.2 and 1.2 m s^−1^ (Davies‐Colley, Payne & van Elswijk, 2000; McCay, 2003), and likely reduce the extent of spore movement. Longer distance dispersal of spore material will, however, occur in the event of extreme storms that damage the vegetation surrounding sporophytes as well as creating turbulence and providing lift (Visher, 1925; Sharpe, 2022).

## III. EVIDENCE OF VECTORED DISPERSAL OF FERN SPORE

### (1) Anemochory

Published studies on atmospheric sampling describing fern spore are few and largely limited to studies on allergens around urban centres. Data suggest that most spore enters spore banks in the immediate deposition zone: spore material from *Botrychium virginianum* (L.) Michx. (height of fertile tissue *ca*. 70 cm) travelled up to 3 m, from *Dennstaedtia punctiloba* (Michx.) T. Moore (height *ca*. 1.0 m) up to 4 m (Peck *et al*., 1990; Penrod & McCormick, 1996), and from *Cyathea arborea* (L.) Sm. (height 8 m) up to 30 m from the plant (Conant, 1978). Turbulence and updrafts around sporophytes likely also drive an unknown proportion of spore to higher altitudes, in particular for taxa such as *Equisetum* where spore have elators (Moran, 2004).

Atmospheric sampling in cities has detected fern spore at least 100 m from source, demonstrating lateral movement distances greater than those described in forest sampling (Camacho & Liston, 2001; Kasprzyk, 2004; Guarín *et al*., 2015). Spore deposition rates in urban areas vary: in south‐eastern Poland, a six‐year pollen sampling study recorded a mean (± SD) of 23 ± 22 fern spore m^−3^ day^−1^ deposited in a volumetric sampler located 12 m from the ground (Kasprzyk, 2004), whereas in Funchal in Madeira, 305 fern spore were deposited per m^3^ over a seven‐year period (Camacho, 2015). In comparison, spore deposition rates in open, semi‐natural areas are relatively higher: a study in northern Argentina recorded a fern spore deposition rate of 275 cm^−2^ year^−1^ of 11 fern species (Torres *et al*., 2014). Unfortunately, few studies on spore deposition record the distances to potential source, size and habit of the potential source sporophyte population (spore load), or the viability of trapped spore.

**Fig. 1:**
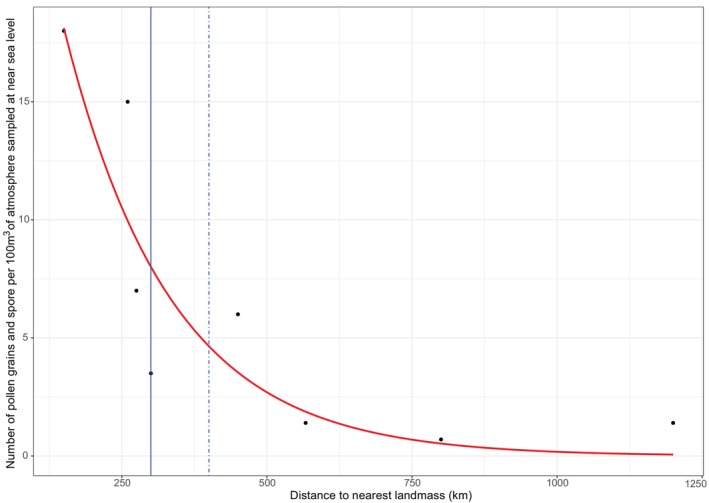
Combined pollen and spore rain concentration (number per 100 m^3^) collected 18 m above sea level on the superstructure of the *M. S. Drottningholm* sailing west from Gothenburg to New York City between 29th May and 7th June 1937, with a negative exponential model fitted (*t*‐value = −10.930, *P* < 0.001). Distance to nearest landmass and concentration values are taken from Fig. 1 and Table 2 of Erdtman (1937) respectively. The solid blue line identifies the distance from shore that the three confirmed *Osmunda* spore were collected; the dot‐dashed line identifies the collection point of a *Dryopteris* and a *Lycopodium clavatum* spore which were possibly present due to sample contamination.

The only offshore atmospheric sampling study of fern spore was undertaken by Erdtman (1937) who sampled near‐continuously at 18 m above sea level for 9 days on an Atlantic crossing during the boreal spring of 1937 [May/June; corresponding to seasonal release of spore (Pettersson, 1940; Suissa, 2022)] and provided a robust proxy for distance from sporophyte source and an indicator of dispersal potential over distances >100 km from source. Erdtman (1937) recorded three *Osmunda* spp. spore up to 300 km off the North American coast at a concentration of *ca*. 1 spore per 526 m^3^ of air. Erdtman's (1937) offshore pollen and spore rain data show that (*i*) fern spore comprise a small percentage of plant spore and pollen rain (maximum 1.3% of sample), (*ii*) sea‐level atmospheric concentration of pollen grain and fern spore decreases exponentially with distance from the coast (Fig. 1), and (*iii*) no fern spore was recorded beyond 300 km from the nearest landmass.

Data for vertical movement of fern spore is limited to two studies and both only collected fern material over land. Systematic atmospheric sampling at different altitudes (100, 500, 1000, 2000 m) above the Netherlands (between the Soesterberg aerodrome and the forested Veluwe hills) in 1936 captured a single fern spore of *Athyrium filix‐femina* at 500 m which was subsequently germinated and developed a gametophyte that self‐fertilised and produced a small sporophyte (van Overeem, 1937; Baas Becking & Raat, 2022). Atmospheric invertebrate sampling by a modified jet aircraft that flew over 187,000 km over the Pacific Ocean in 1960 collected a single fern sporangium at 2400 m above the landmasses of Hawai'i (Gressit *et al*., 1961) but the viability of the spore material was not recorded. Dispersal in the high atmosphere is risky (Tryon, 1970), with these risks loosely parameterised by Gradstein & van Zanten (1999) who established that ultra‐violet (UV) radiation will likely sterilise all but the most common species of spore above 3000 m altitude. Therefore, although Geiger & Ranker (2005) and Geiger *et al*. (2007) hypothesised that the northern subtropical jet‐stream (5,500–17,000 m) might be responsible for delivering spore of ancestral *Dryopteris* taxa to Hawai'i, jet‐stream and high‐altitude dispersal of fern spore is likely unviable for many species.

A key study examining wind as a disperser of ferns identified significant correlations between fern communities across Southern Ocean islands and sea‐surface wind patterns (Muñoz *et al*., 2004). However, subsequent statistical investigation has shown that the Mantel test used is confounded by spatial autocorrelation, and that work is needed to show whether the strength of the correlation between wind connectivity and fern community remains robust when corrected for this error (Guillot & Rousset, 2013).

### (2) Zoochory

Many fern spore have structures (ridges, grooves) that may have a function in epizoochory (Page, 1982). Spore, sporangia, and sporophyte have been collected from the skins, feathers, and fur of salamanders, newts, squirrels, ducks, and the exoskeletons of ants (Page, 1982; Tryon, 1985; Barbé *et al*., 2016; Oralls *et al*., 2016; Coughlan, Kelly & Jansen, 2017). The described zoochory dispersal events of fern spore have been mostly over short to moderate distances; however, birds or bats moving between continental landmasses and islands as well as across archipelagos may provide a regular, direct connection between islands (Wallace, 1880; Cameron *et al*., 2006; Sugita *et al*., 2013; Booth Jones *et al*., 2017; Abeyrama *et al*., 2021). Fern spore has been recovered from a range of seabird feathers including petrels and albatross (Abeyrama *et al*., 2021; J.M.R. Brock, unpublished data). Epizoochory permits the possibility of achieving dispersal over long distances, before the spore detaches when the animal is resting, nesting, or during preening, etc. (Boch *et al*., 2016). For example, the high proportional representation of chlorophyllous‐spored fern species on islands (Mellado‐Mansilla *et al*., in preparation) may be facilitated by spore dispersal in bird feathers.

Endozoochory is another form of zoochory where animals consume fern material, either directly or by secondary consumption, for example where an invertebrate has consumed fern spore and is subsequently eaten by a bat or bird (Daniel, 1979; Brock & Collier, 2020). Fern spore also regularly passes intact through the gut of invertebrates, amphibians, mammals, and birds (Duthie, 1929; Van Tooren & During, 1988; Schneller, 1998; Bråthen *et al*., 2007; Arosa *et al*., 2010; Boch *et al*., 2013, 2016; Sugita *et al*., 2013; Hervías‐Parejo *et al*., 2019; Brock & Collier, 2020; Pauly *et al*., 2024). Endozoochory is limited by gut passage time (Manzano & Malo, 2006), although moderate dispersal distances are possible (Lovas‐Kiss *et al*., 2018, 2019; Hervías‐Parejo *et al*., 2019). For some taxa both endo‐ and epizoochory have been observed: *Isoetes*, with spiny micro‐ and megaspores, will both attach externally to and pass successfully through the gut of animals including waterfowl, muskrat *Ondatra zibethicus* L., reptiles, amphibians, spawning fish, and beaver *Castor canadensis* (Kuhl) as well as the gut of earthworms and snails (Duthie, 1929; Page, 1982; Taylor & Hickey, 1992; Brunton & Britton, 1993, 1999; Caplen & Werth, 2000; Troia, 2016).

**Fig. 2:**
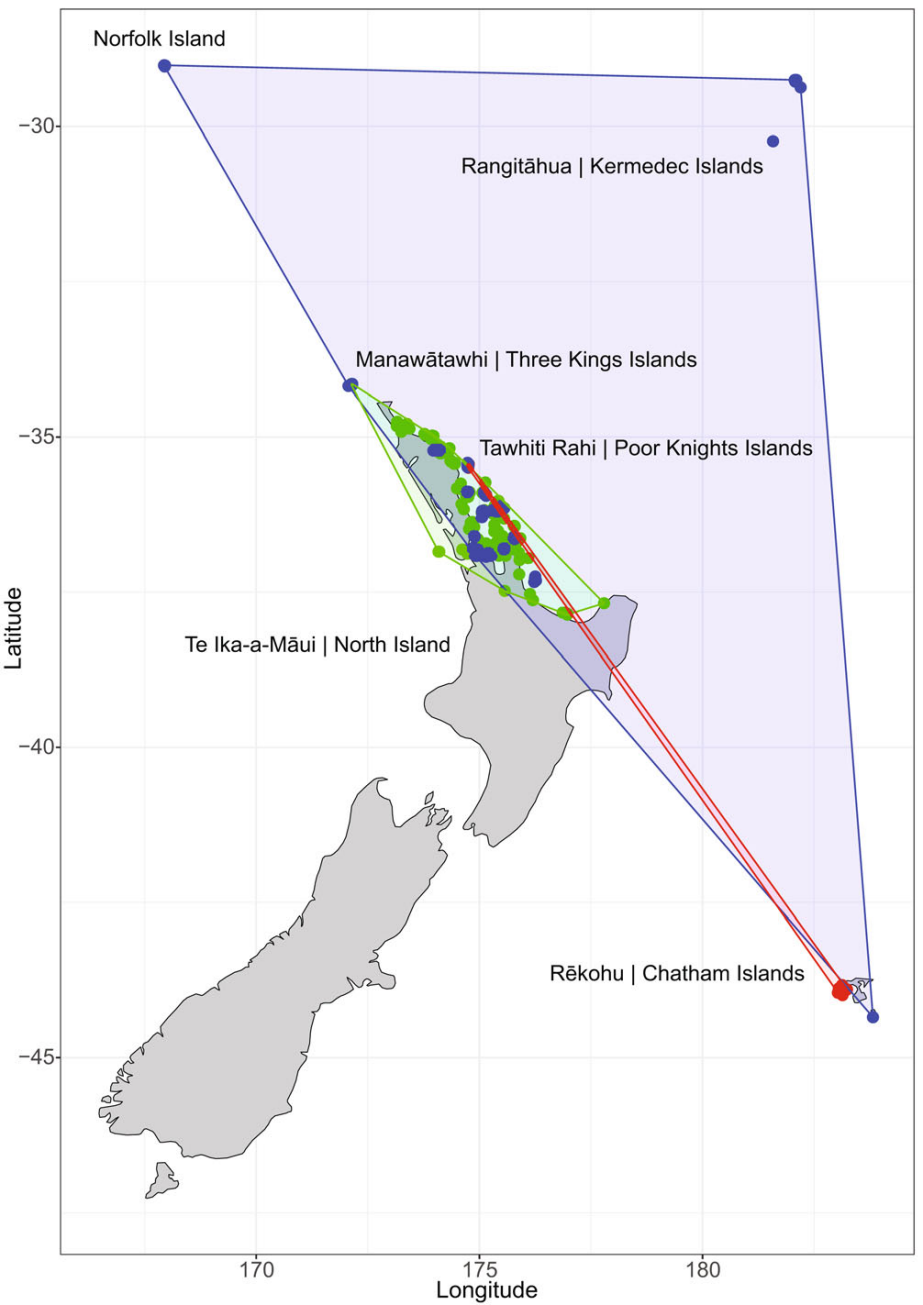
Distribution of seabird colony‐associated fern taxa with low spore loads across New Zealand and nearby islands. Blue = *Austroblechnum norfolkianum*; green = *Asplenium haurakiense*; red = *Asplenium pauperiquitrum*. Norfolk island (34.6 km^2^) is 760 km from New Zealand, Rangitāhua | Kermadec Islands (33.6 km^2^) are 1000 km away, and Rēkohu | Chatham Islands (794 km^2^) are 680 km distant.

Some fern taxa are either dependent on, or restricted to, specific conditions that are engineered by animals. In New Zealand, *Asplenium haurakiense* (Brownsey) Ogle and *Austroblechnum norfolkianum* (Heward) Gasper et V.A.O.Dittrich occur only in coastal forests on nitrogen‐ and phosphorus‐rich soils around seabird burrows, in particular those of petrels (Procellariiformes) (Fig. 2; Wright *et al*., 2010). These small ferns (40 and 90 cm tall respectively) occur sparsely as individuals in the understorey and therefore produce low spore loads. However, petrels carry fern spore in their feathers (as proposed by Wallace, 1880; J.M.R. Brock, unpublished data), as well as in dead‐skin anklets around their legs (Chris Gaskin, Northern New Zealand Seabird Trust, personal communication). Petrels potentially accumulate spore from spore banks in nesting burrow walls and sporophytes whilst moving from their nests to launching sites. The regularity of petrel journeys between conspecific colonies has not been quantified. Although many seabirds are understood to be strongly philopatric (Abeyrama *et al*., 2021), large numbers (hundreds) of non‐breeding juveniles have been recorded seasonally visiting non‐natal conspecific colonies in multiple island and mainland locations across the eastern Atlantic (Munilla *et al*., 2016).

Wallace (1880) described Tahitian petrel *Pseudobulweria rostrata* Peale colonies in Tahiti in dense fern‐dominated vegetation, similar to those colonies of petrels and albatross in Hawai'i and across other islands of the Pacific, and was the first to suggest that birds may carry spore in their feathers. Not only are seabirds driving environmental conditions and disturbance events that support fern‐dominated communities, but by association they are also potentially driving effective dispersal of spore across fern populations of distant island groups (see Box 2 in Gillespie *et al*., 2012; Mason, Baruzzi & Lashley, 2022). For example, a population of the hyper‐endemic, low‐spore‐load *Asplenium pauperequitrum* Brownsey et P.Jackson was discovered on the Chatham Islands 1245 km from the nearest known population on the Poor Knights Islands (Fig. 2; Cameron *et al*., 2006). *Asplenium pauperequitrum* is small (sporophytes 5–10 cm tall), occurs in sparse populations in shaded cliff crevices, and has slowly disarticulating indusium. Buller's shearwater *Puffinus bulleri* (Salvin) inhabit the areas in which both fern populations occur suggesting that this seabird is a potential vector (Cameron *et al*., 2006). The ability for seabirds to connect insular fern communities is of interest in the context of Tryon's (1970) observation of the similarities of the isolated fern communities of Madeira and the Azores, given the similarities in the seabird communities of these archipelagos (Munilla *et al*., 2016).

Zoochory may be particularly important for small ferns with a low spore load that are associated with dense herbaceous vegetation, such as members of the Ophioglossaceae. Although no studies have established post‐zoochorous viability of spore and the impacts of herbivory on plant and population viability, *Botrychium mormo* W.H. Wagner, Jr. is incapable of opening its fertile frond but the same fertile frond is regularly eaten by an un‐named mammal (Wagner *et al*., 1985). Palynological studies have also reported high concentrations of *Ophioglossum* spp. spore in coprolites (Wood *et al*., 2021). Furthermore, birds have been shown to disperse fungal taxa that form mycorrhizal associations with ferns, suggesting that zoochory could support both spore and symbiont dispersal (Caiafa *et al*., 2021). However, not all herbivory will be beneficial, and invertebrate herbivory has also been shown to damage growth of the fertile frond and prevent spore development in *Botrychium multifidum* (Gmel.) Rupr. (Mesipuu, Shefferson & Kull, 2009).

### (3) Hydrochory

There is little published information on fern spore dispersal in water. The most commonly described dispersal vector of aquatic ferns such as *Azolla*, *Salvinia*, *Marsilea*, and *Isoetes* is zoochory, mostly by aquatic animals and humans moving between waterways (Duthie, 1929; Malone & Proctor, 1965; Taylor & Hickey, 1992; Brunton & Britton, 1993, 1999; Caplen & Werth, 2000).

### (4) Summary of potential and constraints of vectors

Wind and animals represent two vectors of fern spore, each with evidence supporting their relative effectiveness. Wind is likely effective at increasing spore concentrations in the soils near the sporophyte and at regularly transporting smaller proportions of spore into the atmosphere. Where deposition occurs before atmospheric damage from UV, temperature, moisture, etc., wind may effectively move spore long distances from the sporophyte. Animals are likely ineffective at moving large quantities of spore and where activity is near to a sporophyte, animal contribution to effective dispersal may be obscured by that of wind dispersal. However, animals moving between locations with similar environmental conditions, even when carrying low spore numbers, may contribute significantly more than wind to the dispersal of both terrestrial and aquatic fern spore. Where wind directionality is a potential constraint to fern spore arriving in suitable habitat or a safe site (e.g. in a complex landscape; Wagner *et al*., 2004), directionality may confer greater value to zoochory where animals share habitat with ferns and in particular where suitable conditions and safe sites are uncommon (Mason *et al*., 2022). Therefore, when considering the potential effectiveness of different fern spore dispersal vectors (see Christ, 1910), we must consider (*i*) the environmental tolerances of fern life stages, and (*ii*) the effects of landscape heterogeneity on spore germination and gametophyte and sporophyte success.

## IV. ENVIRONMENTAL TOLERANCES OF DIFFERENT FERN LIFE STAGES

**Fig. 3:**
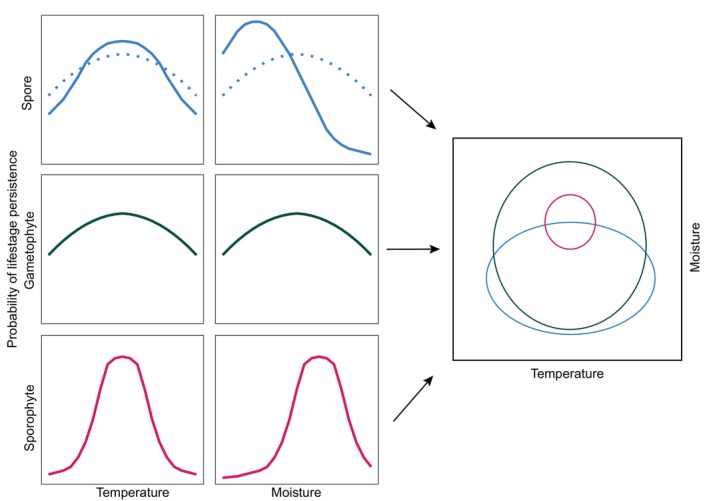
A theoretical model of the environmental filters acting differentially on spore, gametophyte, and sporophyte stages (spore: solid blue line = persistence in spore bank; dotted blue line = spore germination post‐dispersal). The environmental space that describes the spore bank or a location where a spore can land and germinate does not necessarily equate to the environmental space necessary for successful development of a sporophyte.

Safe sites (*sensu* Harper *et al*., 1961) are locations that will support the establishment and growth of successful sporophytes. However, the separate life stages of ferns create a challenge for the safe site concept, as gametophytes appear to have different tolerances relative to sporophytes (Watkins Jr *et al*., 2007b; Nitta *et al*., 2017). Safe sites have been described for specific life stages, for example *Blechnum spicant* L. gametophytes are more likely to occur in areas of ungulate disturbance than in the surrounding forest (Cousens, Lacey & Kelly, 1985). Such a safe site description suggests that fern safe sites can emerge from disturbance events and that spore could either arrive on the soils exposed by ungulate disturbance and germinate, or spore are already present in the soils and germinate in response to the disturbance. Therefore, safe sites for ferns can represent areas of suitable habitat for germination and gametophyte development, or areas where conditions support the persistence of spore in soil until a disturbance event whereupon germination can occur (Fig. 3).

The importance of immediate germination *versus* entering the spore bank will vary according to whether spores are chlorophyllous, achlorophyllous, or cryptochlorophllous (Pence, 2000; Sundue, Vasco & Moran, 2011), and will likely change across a gradient from abundant suitable habitat in a benign environment (*sensu* Cousens *et al*., 1985) to rare suitable habitat in an otherwise hazardous environment (Nobel, 1978; Havanond, Aksornkoae & Nakamura, 1999; Cannicci *et al*., 2018). For example, at the hazardous extremes, sporophytes of the desert fern *Notholaena parryi* D.C.Eaton occur only in association with protective rock piles (Nobel, 1978), *Dryopteris ludoviciana* (Kunze) and *Thelypteris* spp. on crayfish *Procambarus* spp. “chimneys” in a hardwood swamp, and *Acrostichum speciosum* Willd. on lobster *Thalassina anomala* (Herbst) and crab *Neosarmatium* spp. mounds in Malaysian and Sri Lankan mangrove swamps respectively (Havanond *et al*., 1999; Cannicci *et al*., 2018); such locations are unlikely to have, or be able to support, a spore bank and these populations would therefore be reliant on spore rain to these safe sites.

A series of filters on the presence of a sporophyte begin with the requirements of spore and include suitable conditions for either (*i*) spore arrival and germination, or (*ii*) spore arrival and persistence in the spore bank (Dyer & Lindsay, 1992), (*iii*) germination in the event of spore bank disturbance (Cousens *et al*., 1985), and (*iv*) successful reproduction. Requirements for immediate germination of a spore on the surface (i.e. spore arrives at a safe site for gametophytes) will likely differ from the requirements for spore to enter and persist in a spore bank (i.e. habitat suitable for spore but not for germination; Fig. 3). Such a dichotomy will not exist for all fern taxa; for example, spore of *Botrychium* and *Ophioglossum* will germinate and form gametophytes underground (Dyer, 1994) suggesting close overlap between spore and gametophyte requirements (the main limiting factor being the presence of a mycorrhizal fungi partner). Environmental conditions influencing the safe site for spore relate to spore type (achlorophyllous, etc.) and soil conditions that support persistence in the spore bank, while threats include extreme temperatures, marine/brackish ecotones, and waterlogging.

After spore germination, the next filters are on gametophyte requirements and successful reproduction. Gametophytes have been shown to have broader environmental tolerances than sporophytes and have been recorded occurring in locations distant from sporophyte populations (Watkins Jr, Mack & Mulkey, 2007a; Ebihara *et al*., 2013; Brock *et al*., 2019). However, high gametophyte mortality (Cousens, 1981; Gureyeva, 2003) suggests that many gametophyte safe sites do not equate to sporophyte safe sites (Fig. 3). For example, Peck *et al*. (1990) recorded 100% loss of over 6000 gametophytes in a field‐based demographic study. Yet some fern species have not only dispensed with the sporophyte life stage, they occur in gametophyte‐only populations that appear to persist for indefinite periods of time, for example *Vittaria appalachiana* (Farrar & Mickel, 1991; Pinson *et al*., 2017). Gametophytes that take a long time to develop and that may persist near indefinitely in the landscape may in effect establish a gametophyte bank as a spore bank (Testo & Watkins, 2011).

Microsite environmental conditions constrain the development of gametophytes and, in the absence of other drivers, gametophyte size is associated with sex (female, male, or both) and how sex changes over time (Guillon & Fievet, 2003; DeSoto, Quintanilla & Méndez, 2008; Brock *et al*., 2019). Further, many fern species use chemical signalling, including antheridiogens, to control the sex of gametophytes across dense populations (Chiou, Farrar & Ranker, 2002; Gureyeva, 2003). While self‐fertilisation in bisexual gametophytes is possible, fern populations are generally the product of outcrossing showing that sperm transfer between gametophytes has occurred (Haufler & Gastony, 1978; Soltis & Soltis, 1992; Ranker & Geiger, 2008; Sessa *et al*., 2016). In experimental conditions, sperm of *Equisetum* can travel *ca*. 1 m h^−1^ through distilled water for up to 2 h; however, in soils, sperm are unlikely to travel more than 2.5 cm (Bilderback *et al*., 1973; Gómez‐Noguez *et al*., 2017b). Short‐distance (<5 cm) journeys by searching sperm therefore impose minimum gametophyte densities for sporophyte selfing or out‐crossing of *ca*. 509 m^−2^ [either between gametophytes of the same parent sporophyte or different parent sporophytes (Sessa *et al*., 2016; Gómez‐Noguez *et al*., 2017b)].

Sporophytes will develop if conditions are suitable outside the topography of the microsite in which the gametophyte has developed, or if conditions change over time to become more suitable in the case of long‐lived gametophytes (Testo & Watkins, 2011; Pinson *et al*., 2017). Competition from adjacent plants, herbivory, disease, and seasonal stressors will all constrain the potential of a sporophyte to develop successfully to maturity. The key to understanding what patterns of sporophyte occurrence represent in terms of dispersal is that not only are a specific suite of conditions and events necessary for a sporophyte to occur, but it is likely that extensive spore dispersal to a new location will have had to have occurred for a sporophyte to be present.

In summary, spore, gametophyte, and sporophyte environmental requirements are nested and strategically, but not fully, aligned (Dyer & Lindsay, 1992; Rumsey & Sheffield, 1996). Further, gametophytes have greater stress tolerances and have been recorded as responding to different environmental gradients than sporophytes and will likely therefore occur in areas that sporophytes will not (Nitta *et al*., 2017; Pinson *et al*., 2017; Krieg & Chambers, 2022). For these reasons, the likelihood of a single spore arriving in a safe site, germinating and – through selfing or apogamy – establishing a new sporophyte population is low. Spore rain (> one spore) is likely required to escape the various filters on establishing a new population in a location distant from the parent sporophyte.

## V. CONSTRAINTS OF LANDSCAPE HETEROGENEITY ON THE EFFECTIVENESS OF SPORE VECTORS

Landscapes represent complex mosaics of heterogenous abiotic and biotic filters across multiple spatial scales that change over time in response to successional processes and disturbance regimes (Turner *et al*., 1993; Prevedello & Vieira, 2010). The landscape matrix consists of a continuum of matrices from safe (e.g. moist soils) to hazardous (e.g. ocean, desert, ice) to fern spore. Spore arriving at any location across a landscape will either be lost, enter the spore bank, or germinate. An increasingly hazardous matrix with concomitantly decreasing safe site size can be described as an isolation gradient (Tryon, 1970; Fahrig, 2003). The probability of propagule arrival at an increasingly isolated site was modelled by Tischendorf, Bender & Fahrig (2003) who showed that distance to safe site and size of safe site were significant and separate constraints on arrival probability. Further constraints on dispersal are raised by directionality where trends in wind direction (direction of non‐directed propagule movement) significantly reduce the probability of propagules arriving at safe sites in an increasingly fragmented landscape (Wagner *et al*., 2004; Mason *et al*., 2022). As such, the landscape matrix is likely to represent a significant constraint on the relative contribution to effective dispersal of fern spore by different vectors.

Where the landscape is heterogenous and non‐hazardous for fern taxa, the likelihood of multiple sporophytes across the landscape is high (i.e. high spore load), and the availability of safe sites and suitable habitat will also be high. In this scenario, effective local dispersal by wind is likely. However, even when spores are being deposited by wind over areas of land, abiotic conditions and physical constraints on establishing and persisting (biotic filters) will cause the loss of most spore (Hille Ris Lambers *et al*., 2012; Gómez‐Noguez *et al*., 2017b). In a landscape of suitable habitat, wind dispersal will ensure the establishment and maintenance of spore banks and create a source for sporophytes in response to disturbance regimes.

**Fig. 4:**
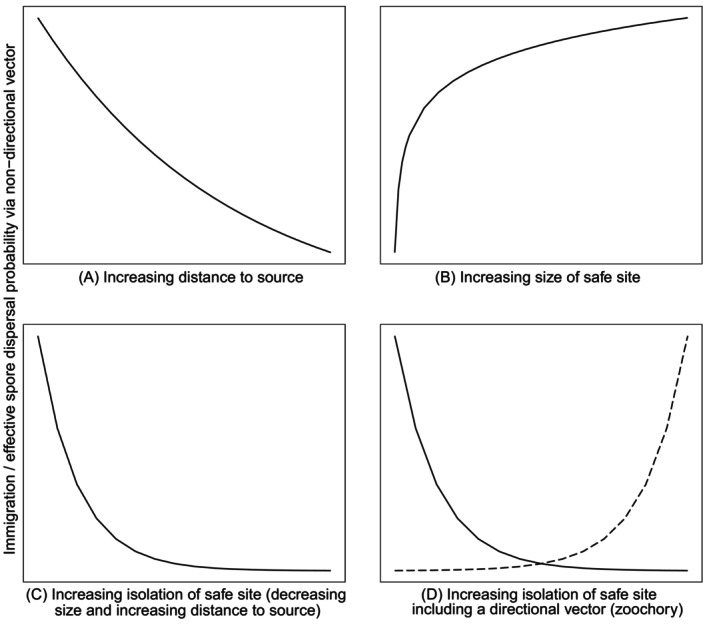
Modelled immigration probability of propagules over (A) increasing distance, and (B) to safe sites of increasing size (adapted from Tischendorf *et al*., 2003). Predicted relationships between immigration probability along (C) an increasing gradient of isolation, that is an interaction of increasing hazardous matrix around safe site (distance to and size of safe site), and (D) considering passive directed dispersal by zoochory (dashed line).

Where the landscape matrix is hazardous to ferns (ocean, desert), effective dispersal events are considerably more constrained, as sporophytes are present at relatively lower densities and therefore spore load is much reduced. With safe sites and suitable habitat increasingly limited, dispersal success by wind decreases exponentially (Fig. 4; Erdtman, 1937; Tischendorf *et al*., 2003; Wagner *et al*., 2004) and multiple viable spore of the same taxa arriving in close adjacency will be extremely rare (Peck *et al*., 1990; Gradstein & van Zanten, 1999). There is likely an interaction between island size and relative isolation of island (Fig. 4C; Tryon, 1970; Fahrig, 2003; Tischendorf *et al*., 2003), not only on species richness and numbers of endemic taxa, but also on the likely effective vector across insular fern meta‐populations.

Isolation, the interaction between size of target and distance to target or density of targets, is likely a key constraint on fern dispersal (Tryon, 1970; Fahrig, 2003; Tischendorf *et al*., 2003). Ferns that establish on elevated surfaces (lobster mounds) in mangrove swamps are using small habitats within a hazardous matrix, but the density of lobster mounds (74–98 per 50 m^2^) means that safe sites are not isolated and fern populations are likely maintained by non‐directed wind dispersal (Havanond *et al*., 1999). Conversely, an archipelago such as Hawai'i, which has a larger area but is over 3600 km from the nearest landmass, means that non‐directed dispersal by wind to these islands is improbable (see constraints of anemochory above). Any fern taxa on Hawai'i that are shared by other landmasses, in particular other Pacific Island groups, are more likely connected by directed dispersal (depending on spore load and abiotic constraints; Mason *et al*., 2022). Although large landmasses with a hazardous matrix would represent a large target for immigrating spore, the entire landmass is unlikely to be suitable for fern establishment, and this heterogeneity will present a hazard to spore (Fahrig, 2003; Heidrich *et al*., 2020). Therefore, effective dispersal between fern meta‐populations across even large islands may be most effective by targeted vectors. Where dispersal events to islands drive speciation – rather than a new population of a cosmopolitan taxa (rare *versus* regular dispersal) – rare or infrequent dispersal events of a few spore might also be driven by zoochory [for example, the wrecking or stranding of, or infrequent visitation by, birds (Kinsky, 1968; Harrison, 1990; Faria *et al*., 2014; Shiomi, 2023)].

If a population of birds, for example, shares the niche of a given fern taxa (particularly one with a small spore load), dispersal across and between islands *via* zoochory is likely to be more effective than by wind (Oralls *et al*., 2016; Mason *et al*., 2022), and may support meta‐populations and similar fern communities across otherwise isolated islands (Török *et al*., 2020). This may be of particular relevance for chlorophyllous‐spored ferns, with, for example, spore being dispersed between isolated island habitats in the feathers of a canopy roosting or wetland bird (Mellado‐Mansilla *et al*., 2022, in preparation). Likewise in other hazardous matrices such as arid savannahs and deserts that support fern populations, mammal and reptile movements may have a similar function to amphibians in forests and vector spore to safe sites and suitable habitat (Oralls *et al*., 2016). Although wind may deposit spore in suitable habitat that eventually, after a disturbance event that triggers spore germination, drives the establishment of a new sporophyte, animals are more likely to deposit spore more regularly in safe sites across a hazardous matrix (Mason *et al*., 2022).

While Muñoz *et al*. (2004) correlated patterns of wind resistance to insular fern communities across the Southern Ocean (confounded by spatial autocorrelation; Guillot & Rousset, 2013), these same islands support breeding populations of seabirds that use wind to migrate and disperse to different breeding colonies (Kogure *et al*., 2016; Richardson, Wakefield & Phillips, 2018; Abeyrama *et al*., 2021). It is possible therefore that seabirds may be driving spore‐dispersed plants across insular fern communities, and that isolation alone will not consistently predict patterns of fern endemism and richness, since connectivity by zoochory may reduce the constraints of island size and physical isolation. Perhaps the migration of *Dryopteris* to Hawai'i from south‐east Asia (as identified by Geiger & Ranker, 2005) was mediated by seabird movement across the western and central Pacific. Associations between specific animals and the ferns that share niche spaces may explain the patterns observed by Tryon (1966) that only a subset of ferns from South America and Africa occur on Tristan da Cunha.

Safe sites in a heterogenous landscape may also result from animal behaviour and mutualisms. The sites are not only shared by fern and animal species, but are established by animal activity driving suitable conditions for ferns through disturbance, for example seabirds burrowing in forest soils to breed (Roberts, Duncan & Wilson, 2007; Bellvé, 2023), and animals defecating ammonium which stimulates *Botrychium dissectum* Spreng. gametophyte development (Melan & Whittier, 1990). The occurrence of *Asplenium obtusatum* G.Forst. in association with insular seabird colonies around the Southern Hemisphere may be a result of habitat creation and dispersal by seabirds and deserves investigation. Some ferns also have mutualisms with mycorrhizal fungi, and require fungi to fully develop a gametophyte and sporophyte (Winther & Friedman 2007). Animals have the potential to disperse mycorrhizal fungi and the potential significance of zoochory for effective fern dispersal of taxa with mycorrhizal requirements is clear (Boast *et al*., 2018; Correia *et al*., 2019; Wood *et al*., 2020; Caiafa *et al*., 2021).

## VI. CONCLUSIONS

(1) The presence of a fern sporophyte in the landscape is constrained by several factors, including the presence of suitable abiotic and biotic conditions, dispersal, the presence of a spore bank and specific disturbance regime, and the ability to reproduce successfully and produce viable sporophytes in a given location.
(2) Patterns of fern distribution historically used to infer fern dispersal, meta‐population connectivity, and speciation are confounded by the effects of spore load, vector effectiveness, the landscape matrix, and the isolation of safe sites. Where the landscape matrix is unsuitable for spore, the requirement of spore dispersal to be targeted increases significantly.
(3) Wind dispersal of fern spore is likely to be highly effective at building up extensive spore banks across the landscape, and this process may drive persistence of fern taxa in landscapes across time. Animal dispersal potentially enables specific dispersal events to new safe sites (passive directed dispersal), particularly where the matrix is hazardous. The proposal that animal dispersal is significant to fern distribution across a fragmented landscape may also be applied to other spore‐dispersed taxa such as bryophytes, building on the work of Chmielewski & Eppley (2019), de Regnier, Brock & Gaskett (2024), and Osorio‐Zuñiga, Fontúrbel & Rydin (2014).
(4) The history of fern dispersal science is marked by arguments for and against wind as the primary vector, but the pro‐wind narrative has persisted, largely without empirical support. Assumptions around fern dispersal have driven circular arguments in the literature such that the presence of similar sporophytes across distant islands must mean connectedness rather than crypsis, cosmopolitan taxa must infer ready wind dispersal, and that ready‐wind dispersal infers connectedness. The evidence reviewed here suggests that single spore dispersal *via* wind as a method of successfully colonising isolated, remote locations is improbable and that abiotic and biotic filtering should be considered when discussing such pivotal events.
(5) This review builds on the hypotheses of early biogeographers and provides a possible answer to the question that plagued scientists like Wallace (1880) and Ridley (1930): if all ferns are readily dispersed, then why do taxa not occur in all suitable conditions? The evidence suggests that disjunct populations of fern taxa are not inherently indicators of wind dispersal and that the most effective dispersal of fern spore across a fragmented landscape may be by animals.
